# Utility of ultra-rapid real-time PCR for detection and prevalence of *Rickettsia* spp. in ticks

**DOI:** 10.1186/s12917-022-03311-7

**Published:** 2022-05-27

**Authors:** A-Tai Truong, Bo-Ram Yun, Mi-Sun Yoo, Jiyeon Lim, Subin Min, Soon-Seek Yoon, Young-Min Yun, Jong-Taek Kim, Yun Sang Cho

**Affiliations:** 1 Parasitic and Honeybee Disease Laboratory, Bacterial and Parasitic Disease Division, Department of Animal & Plant Health Research, Animal and Plant Quarantine Agency, Gimcheon, 39660 Republic of Korea; 2 Faculty of Biotechnology, Thai Nguyen University of Sciences, Thai Nguyen, Vietnam; 3 Department of Veterinary Internal Medicine, Wildlife Rescue Center, College of Veterinary Medicine, Jeju National University, Jeju, 63243 Republic of Korea; 4 Wildlife Rescue Center, College of Veterinary Medicine, Kangwon National University, Chuncheon, 24341 Republic of Korea

**Keywords:** *Rickettsia*, Republic of Korea, Ultra-rapid real-time PCR, Ticks

## Abstract

**Background:**

*Rickettsia* spp. are important tick-borne pathogens that cause various human and animal diseases worldwide. A tool for rapid and accurate detection of the pathogens from its vectors is necessary for prevention of Rickettsioses propagation in humans and animals, which are infested by ticks. Therefore, this study was conducted to evaluate a molecular tool, ultra-rapid real-time PCR (UR-qPCR), for rapid and accurate detection of *Rickettsia* spp. from 5644 ticks in 408 pools collected from livestock and their surrounding environments in Gangwon and Jeju province in South Korea.

**Results:**

The UR-qPCR of *Rickettsia* DNA showed a limit of detection of 2.72 × 10^1^ copies of *Rickettsia* DNA and no cross reaction with other tick-borne pathogens, namely *Anaplasma phagocytophilum*, *Ehrlichia chaffeensis*, *E. canis*, *Toxoplasma gondii*, and *Borrelia burgdorferi*. In addition, the PCR assay also showed possibility of various *Rickettsia* species detection including *R. monacensis,* “*Candidatus* R. longicornii”*, R. japonica, R. roultii,* and *R. tamurae.* The collected ticks were identified with major species belonged to *Haemaphysalis longicornis* (81.62%), followed by *H. flava* (15.19%)*,* and *Ixodes nipponensis* (3.19%). *Rickettsia* detection from tick samples using the UR-qPCR showed that the minimum infection rate (MIR) of *Rickettsia* in collected ticks was 1.24‰ and that all positive pools contained *H. longicornis,* equal to the MIR of 1.39‰ of this species. Additionally, MIR of *Rickettsia* spp. detected in ticks collected in Gangwon and Jeju was 1.53‰ and 0.84‰, respectively. Furthermore, the sequencing results of the 17 kDa protein antigen gene and *ompA* gene showed that *Rickettsia* spp. sequences from all pools were related to “*Candidatus* R. longicornii” and “*Candidatus* R. jingxinensis”.

**Conclusions:**

The UR-qPCR system was demonstrated to be useful tool for accurate and rapid detection of *Rickettsia* from its vector, ixodid ticks, within 20 min. The data on *Rickettsia* spp. in ticks detected in this study provide useful information on the distribution of *Rickettsia* in previously unstudied Korean provinces, which are important for the prevention and control of the spread of rickettsioses in both animals and humans in the country.

## Background

The obligate intracellular bacteria of *Rickettsia* genus are commonly harboured and transmitted by arthropods, mainly ticks [1–3], some of the bacteria cause Rickettsioses in animals and humans with mild to life-threatening consequences [4]. *Rickettsia* and the related tick vectors have been reported in different countries. For example, four subspecies of *R. conorii,* the cause Mediterranean spotted fever in Europe, were mainly found in *Rhipicephalus sanguineus* and *Rh. pumilio* ticks [5, 6], the Japan spotted fever group (*R. tamurae*, *R. japonica*, *R. raoultii*, and *Candidatus* R. principis) was detected in *Haemaphysalis* and *Amblyomma* ticks in Japan [7], and *R. raoultii* was prevalent in *Dermacentor nuttalli* and *Dermacentor silvarum* ticks in China [8, 9]. The information of *Rickettsia* species and related tick species in a particular region is important to identify the risk of Rickettsioses transmission via tick bite.

Polymerase chain reaction (PCR) has been used as a sensitive and specific tool for the rapid detection of *Rickettsia* from both ticks and patients [10–14], and species identification of *Rickettsia* was done by sequencing analysis of various genes, such as the rickettsial citrate synthase gene (*gltA*) [15], SFGR-specific 190 kDa outer membrane protein A gene (*ompA*) [16], outer-membrane protein rOmpB (OmpB) [17], surface cell antigen gene “gene D” (Sca4) [18, 19], and the genus-specific 17 kDa outer membrane antigen gene [20]. Afterwards, real-time PCR was demonstrated to be more sensitive and rapid compared to conventional nested PCR for *Rickettsia* detection, and become an important tool for screening of *Rickettsia* from its natural reservoirs or vectors [21–23]. However, the current *Rickettsia* detection real-time PCRs are still time-consuming systems. A new chip-based PCR system named ultra-rapid real-time PCR (UR-qPCR) has been developed, which has optimal thermal transfer with chip-based reaction that reduces turnaround time. In addition, this small-footprint device with low power consumption make it possible for point-of-care testing application. It has been shown to be useful for the sensitive and rapid detection of honeybee pathogens on-site [24–26]. Therefore, the UR-qPCR could be a useful tool for rapid detection of *Rickettsia* from ticks.

In Korea, Rickettsioses in humans have been reported since 2006 [27, 28]. Thereafter, the role of ticks in carrying and transmission of *Rickettsia* spp. to humans was also demonstrated [29–34]. Monitoring of *Rickettsia* in ticks was done in northern and western regions of the country and showed that “*Candidatus* R. longicornii” was the most prevalent *Rickettsia* species carried by ticks [30, 35, 36]. However, the information of *Rickettsia* harboured by ticks in other regions of the country is still remained uncharacterized.

Accordingly, this study was conducted to examine the ability of the UR-qPCR system for detection of *Rickettsia* in ticks collected from wild animals and livestock in two provinces: Gangwon and Jeju, located in northeastern and southern region of South Korea, respectively. Sequencing and phylogenetic analyses of detected *Rickettsia* spp. were done using the 17 kDa protein antigen and *ompA* genes.

## Results

### Sensitivity and specificity of *Rickettsia* UR-qPCR

**Fig. 1:**
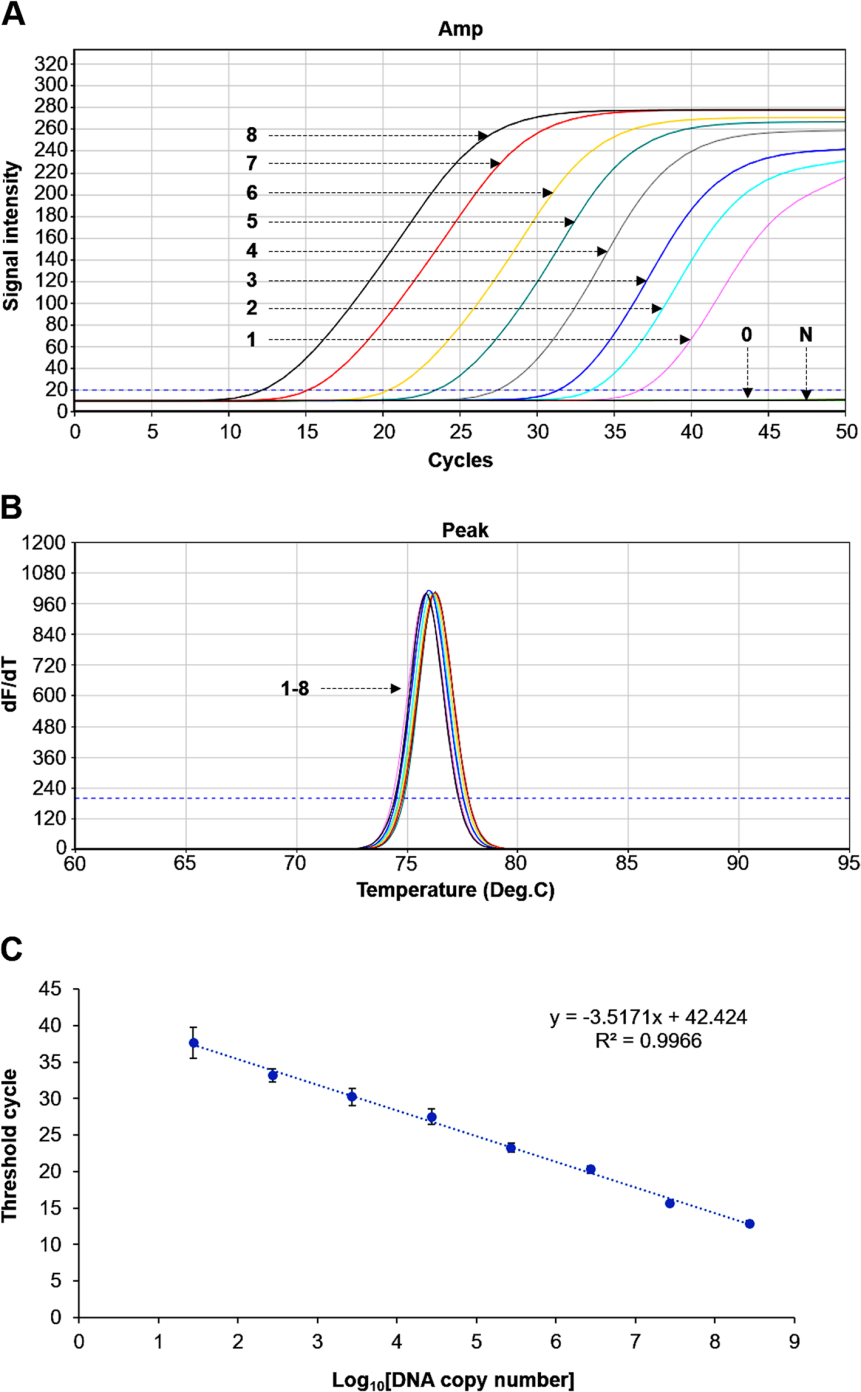
Sensitivity of detection of *Rickettsia* species using ultra-rapid real-time PCR (UR-qPCR). Amplification curves of UR-qPCR using 2.72 × 10^8^ to 2.72 × 10^0^ copies of *Rickettsia* DNA (denoted by number 8 to 0; **A**). The melting curves show *Rickettsia* detection is possible from 2.72 × 10^8^ to 2.72 × 10^1^ copies of target DNA (number 8–1; **B**). “N” is the negative control without a DNA template. Linear regression representing the relationship between cycle threshold of amplification (Ct value) and initial DNA copy number (**C**) was established by amplifying 10-fold dilutions of *Rickettsia* DNA from 2.72 × 10^8^ to 2.72 × 10^1^ DNA copies in triplicate

**Fig. 2:**
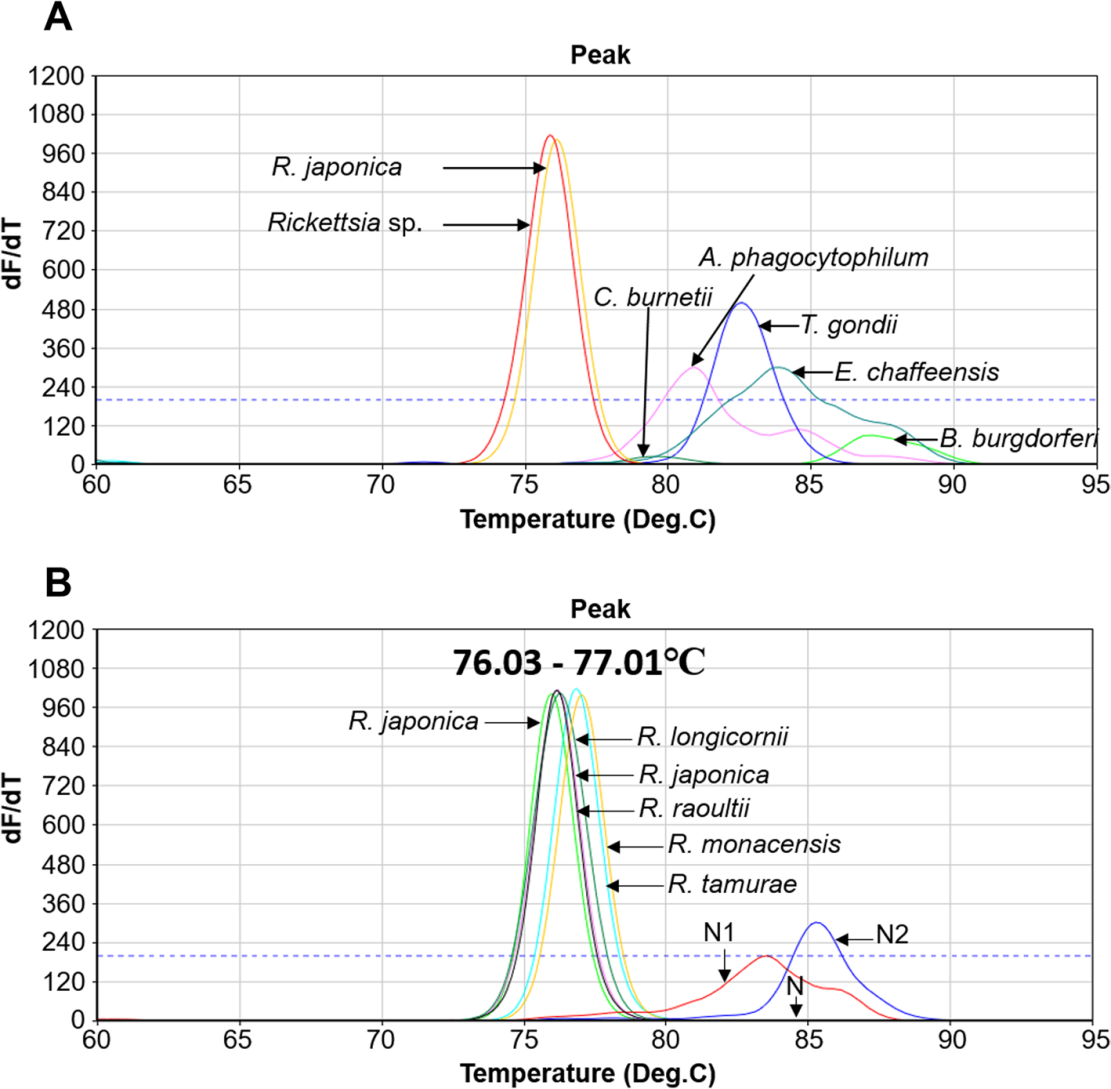
Specificity of detection of *Rickettsia* species using ultra-rapid real-time PCR (UR-qPCR). The specificity of *Rickettsia* UR-qPCR is demonstrated by different melting temperatures observed when amplifying *Rickettsia japonica* recombinant DNA, *Rickettsia* sp. DNA from total nucleic acids isolated from tick sample, and the DNA of other common tick-borne pathogens, namely *Anaplasma phagocytophilum*, *Ehrlichia chaffeensis*, *E. canis*, *Toxoplasma gondii*, *Borrelia burgdorferi, Coxiella burnetii,* and samples with no DNA template (**A**)*.* The melting temperature ranging from 76.03 °C to 77.01 °C were seen from detection PCR using DNA template of five different *Rickettsia* species (**B**). “N1” and “N2” are negative result using total nucleic acids isolated from two tick pools, and “N” is negative control without DNA template

Amplification using serially diluted recombinant DNA showed a limit of detection of 2.72 × 10^1^ copies of *Rickettsia* DNA (Fig. 1A and B). The linear regression representing the relationship between initial DNA copy and cycle threshold (Ct) of amplification was established from triplicate PCR reactions, *y* = − 3.5171*x* + 42.424; R^2^ = 0.9966, where *x* and *y* are the log_10_ DNA copy number and Ct value, respectively (Fig. 1C). The amplification efficiency calculated from the slope of the standard curve (E = 10^(− 1/slope)^-1) was 92.45%. Furthermore, the peaks of melting temperature of amplification (Fig. 2A) showed that the UR-qPCR can be used for specific detection of *Rickettsia* among the tested DNA templates originating from other tick-borne pathogens, namely *Anaplasma phagocytophilum*, *Ehrlichia chaffeensis*, *E. canis*, *Toxoplasma gondii*, *Coxiella burnetii,* and *Borrelia burgdorferi.* In addition, the melting peaks of five different *Rickettsia* spp. (*R. japonica, R. roultii,* “*Candidatus* R. longicornii”, *R. monacensis,* and *R. tamurae*) detection were not greatly different, ranging from 76.03 °C to 77.01 °C (Fig. 2B). The result demonstrated that the UR-qPCR assay can be used as a molecular tool for detection of various *Rickettsia* species.

### Prevalence of *Rickettsia* species in ticks

The tick species identified from the highest number of pools were *Haemaphysalis longicornis* (333/408 pools; 81.62%), followed by *H. flava* (62/408 pools; 15.19%), and *Ixodes nipponensis* (13/408 pools; 3.19%); *H. longicornis* and *H. flava* were present in samples collected from both Gangwon and Jeju provinces, whereas *I. nipponensis* was only detected in samples collected from Gangwon province.

Moreover, among the three most common tick species identified, only *H. longicornis* from both provinces harboured *Rickettsia* spp. The minimum infection rate (MIR) in Gangwon province as determined by ITS DNA detection using UR-qPCR and by 17 kDa protein antigen and *ompA* gene detection using conventional nested PCR was 1.53‰ (5/408 pools), 1.22‰ (4/408 pools), and 1.53‰ (5/408 pools), respectively. In Jeju province, the MIR was 0.84‰ (2/408 pools), 2.53‰ (6/408 pools), and 2.53‰ (6/408 pools) as detected by ITS, 17 kDa protein antigen gene, and *ompA* detection, respectively (Table 1).

**Table 1:** Detection rates of *Rickettsia* spp. from different tick species collected in Gangwon and Jeju provinces

Province	Tick species	Life stage	Number of ticks	Number of positive pools (MIR)		
				ITS	17 kDa	***ompA***
Gangwon	*Haemaphysalis longicornis*	Larva	2764	0	0	0
		Nymph	50	1 (20.00)	1 (20.00)	1 (20.00)
		Male adults	38	2 (52.63)	2 (52.63)	2 (52.63)
		Female adults	240	2 (8.33)	1 (4.17)	2 (8.33)
	*Haemaphysalis flava*	Larva	0	0	0	0
		Nymph	93	0	0	0
		Male adults	28	0	0	0
		Female adults	10	0	0	0
	*Ixodes nipponensis*	Larva	0	0	0	0
		Nymph	16	0	0	0
		Male adults	3	0	0	0
		Female adults	30	0	0	0
	***Subtotal***		***3272***	***5 (1.53)***	***4 (1.22)***	***5 (1.53)***
Jeju	*Haemaphysalis longicornis*	Larva	1470	0	0	0
		Nymph	158	0	1 (6.33)	1 (6.33)
		Male adults	99	1 (10.10)	1 (10.10)	1 (10.10)
		Female adults	208	1 (4.81)	4 (19.23)	4 (19.23)
	*Haemaphysalis flava*	Larva	0	0	0	0
		Nymph	368	0	0	0
		Male adults	30	0	0	0
		Female adults	39	0	0	0
	***Subtotal***		***2372***	***2 (0.84)***	***6 (2.53)***	***6 (2.53)***
Total			**5644**	**7 (1.24)**	**10 (1.77)**	**11 (1.95)**

*MIR* Minimum infection rate depicted in ‰, *ITS* Internal transcribed spacer. ITS region of *Rickettsia* was detected by UR-qPCR, and other two genes (17 kDa protein antigen and *ompA*) were detected by conventional nested PCR

The overall MIR was 1.24‰ (7/408 pools), 1.77‰ (10/408 pools), and 1.95‰ (11/408 pools) for the detection methods targeting ITS, 17 kDa protein antigen gene, and *ompA* gene, respectively (Table 1). The MIR according to the developmental stages of the infected tick species ranged from 4.81–9.62‰ for nymphs, 21.89‰ for adult males, and 6.70–13.39‰ for adult females; the prevalence was 0% for larvae.

### Sequencing and phylogenetic analysis

**Fig. 3:**
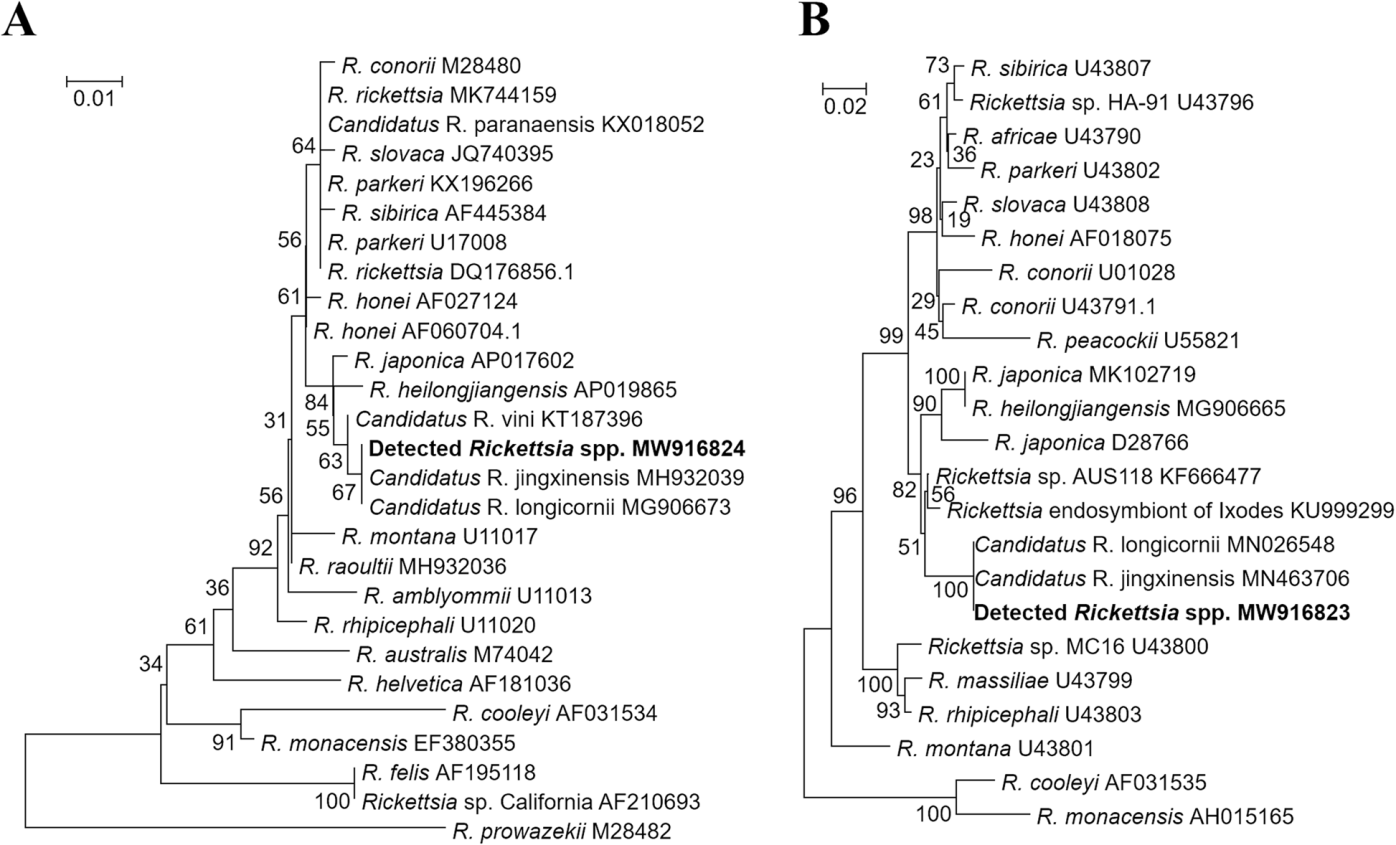
Phylogenetic trees of *Rickettsia* species. The trees were created based on the 410 bp 17 kDa protein antigen gene (**A**) and the 488 bp *ompA* gene (**B**) of *Rickettsia* species using the neighbour-joining method and bootstrap analysis (1000 reiterations) carried out according to the Kimura 2-parameter method in MEGA7 software

Among the 10 pools, generated sequences of the 17 kDa protein antigen gene were 100.00% identical to each other (NCBI accession No.: MW916824) and had 100.00% identity with NCBI deposited sequences of “*Candidatus* R. longicornii” and “*Candidatus* R. jingxinensis” that had been detected in *H. longicornis* ticks in Korea and China, respectively. Additionally, the sequences of the *ompA* gene (NCBI accession No.: MW916823) were 100.00% identical among all 11 pools and showed 100.00% identity to sequences of “*Candidatus* R. longicornii” and “*Candidatus* R. jingxinensis” detected from *H. longicornis* ticks in Korea and China, respectively. Phylogenetic analysis of the two genes showed that the detected *Rickettsia* spp. clustered with “*Candidatus* R. jingxinensis” and “*Candidatus* R. longicornii” when compared to *Rickettsia* spp. originating from other countries (Fig. 3).

## Discussion

Screening of *Rickettsia* was done from ticks collected from Gangwon province, located in northeastern Korea, and from Jeju island, located in southern Korea. Only *H. longicornis* ticks were found to be the vector of *Rickettsia* with positive result of 11/408 pools detected by *ompA* gene. The detected *Rickettsia* spp. all showed 100% sequence similarity to “*Candidatus* R. longicornii” and “*Candidatus* R. jingxinensis”. The result of this study was consistent with previous report [35] that only “*Candidatus* R. longicornii” was detected in *H. longicornis* in the northern area near the demilitarized zone in South Korea. However, various *Rickettsia* species were detected in *H. longicornis* ticks in other regions of the country, such as *R. japonica, R. heilongjiangensis*, and *R. monacensis* in northwestern and southwestern provinces [30], “*Candidatus* R. longicornii” and *R. koreansis* in midwestern region [36]. *Rickettsia* spp. were also minorly detected in *H. flava* and *I. nipponensis* in these regions. The result of *Rickettsia* detection in this study could be important to fulfil the knowledge of distribution of *Rickettsia* and its vectors in the country.

“*Candidatus* R. jingxinensis” and “*Candidatus* R. longicornii” were detected mainly in China and South Korea, in which the name “*Candidatus* R. jingxinensis” was first proposed as a potential new species based on the analysis of *gltA* and *ompA* genes [8] for a *Rickettsia* sp. detected in *H. longicornis* ticks collected at Jingxin town in Jinlin province, China. This species was then identified in *Rhipicephalus microplus* tick in China and in *H. longicornis* in South Korea [37, 38]. The other proposed species, “*Candidatus* R. longicornii”, was introduced based on the analysis of *rrs, gltA, ompA, ompB*, and *sca4* genes [35]. This species was also prevalently detected in *H. longicornis* tick distributed in South Korea, and China [38–40]. However, phylogenetic analysis using the 17 kDa protein antigen and *ompA* genes in this study showed that the two proposed species have a close phylogenetic relationship and the two genes of “*Candidatus* R. jingxinensis” and “*Candidatus* R. longicornii” shared 100% identity. In addition, the *gltA* gene of the two species was also demonstrated to be 100% identical to each other [37]. Therefore, “*Candidatus* R. jingxinensis” and “*Candidatus* R. longicornii” could be the only one species, and according to the prevalence of the organisms detected in *H. longicornis* tick, the only name “*Candidatus* R. longicornii” should be used for the *Rickettsia* species.

The distribution of tick species identified from Gangwon and Jeju provinces was the same as that reported in other provinces; *H. longicornis* was the most abundant species among the three most common tick species in Korea - *H. longicornis, H. flava,* and *I. nipponensis* [41, 42]. The *H. longicornis* tick is a common parasite of livestock, wild animals, and humans, and it is distributed in ten countries including eastern Asia, the USA, Australia, and New Zealand [35, 43]. *H. longicornis* ticks were known to be vectors of various diseases including rickettsioses [43–45].

The loop-mediated isothermal amplification (LAMP) assay was developed for rapid detection of *Rickettsia* from ticks within 30 min [46], using the crude DNA prepared by heating method [47] the LAMP was demonstrated to useful for on-site detection of *Rickettsia* from vectors. However, using hydroxynaphthol blue as a colorimetric component for visual detection by naked eye in LAMP could make a challenge for different readers, and therefore the results need to be confirmed in electrophoresis [46]. The UR-qPCR evaluated in this study showed possibility of various *Rickettsia* species detection within 20 min could address the disadvantages of LAMP for accurate and rapid detection of *Rickettsia*. Using the crude preparation of DNA [47] the UR-qPCR could be used for on-site screening of *Rickettsia* from ticks.

## Conclusions

In this study, a molecular tool UR-qPCR for the rapid detection of *Rickettsia* spp. in ticks was initially examined. The PCR system showed a limit detection of around 27.2 copies of *Rickettsia* DNA within around 20 min. The possibility of various *Rickettsia* species detection was confirmed, and the usefulness of *Rickettsia* spp. detection was also demonstrated from tick samples. The rapidity and mobility of this PCR system could be important to develop a molecular tool for on-site detection of *Rickettsia* sp. from its vectors. Additionally, the prevalence data on *Rickettsia* spp. identified in ticks collected from livestock and wild animals in the Gangwon and Jeju provinces provide useful information on *Rickettsia* distribution in previously unstudied Korean provinces; this is important for the prevention and control of the spread of rickettsioses in both animals and humans in the country.

## Methods

### Tick sample collection

A total of 5644 larval, nymphal, and adult ticks were collected from livestock, wild animals, and vegetation surrounding the farms or living areas of wild animal in the Gangwon and Jeju provinces in Korea between August and November in 2019. Species of ticks were identified by their morphological characteristics using a stereomicroscope (Discovery.V8; ZEISS, Oberkochen, Germany) and the standard illustrated taxonomic key [48]. After identification of species, the samples were pooled for the living stages of the same species collected from the same site. Each pool contained 1, 1 to 10, and 1 to 50 individuals of adult, nymph, and larvae, respectively. Totally, 408 pools were acquired, in which 235 pools were collected from Gangwon and designated as 19 M1 to 19 M235, while 173 pools were collected from Jeju and labelled as 19 T1 to 19 T173. The samples were then preserved in 70% ethanol and stored at − 80 °C until further analysis.

### Nucleic acid extraction from ticks

Briefly, ticks from each pool were washed three times using the UltraPure™ distilled water (Thermo Fisher Scientific, Massachusetts, USA), and were placed in a tissue homogeniser with steel beads 2.381 mm diameter (SNC, Hanam, Korea). After adding 600 μl of PBS solution, the sample was homogenised using a Precellys 24 Tissue Homogeniser (Bertin Instruments, Montigny-le-Bretonneux, France). Then 300 μl of the homogenate was transferred to a new tube that contained 300 μl of lysis buffer and 30 μl of proteinase K solution. The mixture was incubated at 56 °C for 10 min and the total nucleic acid was extracted using the Maxwell® RSC Viral Total Nucleic Acid Purification Kit on the automated Maxwell® RSC Instrument (Promega, Madison, WI, USA) according to the manufacturer’s instructions. Finally, 50 μl of total nucleic acid was acquired from each sample.

### PCR performance

The internal transcribed spacer (ITS) region of *Rickettsia* spp. was targeted for detection in tick samples using the GENECHECKER® UF-150 UR-qPCR system (Genesystem Co., Ltd., Daejeon, Korea) and 2× Rapi: Detect™ Master mix with dye (SYBR green, Cat. No.: 9799100100; Genesystem Co.). The 10 μl reaction mix consisted of 1 μl (10 pmol) of each primer (ITS-F/R; Table 2), 5 μl of PCR premix, and 3 μl of total nucleic acid. The PCR conditions were examined at different annealing temperature from 52 °C to 66 °C to select the optimal condition for specific and sensitive detection of *Rickettsia* spp., final PCR conditions are shown in Table 2. Recombinant ITS DNA of *R. japonica* (NCBI accession number CP047359) was used for optimizing PCR conditions and was used as positive control for *Rickettsia* spp. detection from tick samples, and no DNA template was used in negative control.

**Table 2:** Primers used for detection and sequencing of *Rickettsia* spp. from ticks

Primer name	Sequence (5′-3′)	Target gene (bp)	Cycling conditions	Reference
ITS-F	GATAGGTCGGGTGTGGAAG	ITS, 388	50 cycles, 95 °C (4 s) -64 °C (4 s)-72 °C (4 s)	[49]
ITS-R	TCGGGATGGGATCGTGTG			
Rr17k. 1p	TTTACAAAATTCTAAAAACCAT	17 kDa protein antigen, 539	35 cycles, 95 °C (30 s)-47 °C (30 s)-72 °C (1 min)	[50]
Rr17k. 539n	TCAATTCACAACTTGCCATT			
Rr17k. 90p	GCTCTTGCAACTTCTATGTT	17 kDa protein antigen, 450	35 cycles, 95 °C (30 s)-52 °C (30 s)-72 °C (1 min)	
Rr17k. 539n	TCAATTCACAACTTGCCATT			
Rr190k. 71p	TGGCGAATATTTCTCCAAAA	*ompA*, 650	35 cycles, 95 °C (30 s)-49 °C (30 s)-72 °C (1 min)	[50]
Rr190k. 720n	TGCATTTGTATTACCTATTGT			
Rr190k. 71p	TGGCGAATATTTCTCCAAAA	*ompA*, 532	35 cycles, 95 °C (30 s)-52 °C (30 s)-72 °C (1 min)	[50]
Rr190k. 602n	AGTGCAGCATTCGCTCCCCCT			[51]

Species identification was performed by nested PCR using the Mastercycler® X50s conventional PCR system (Eppendorf, Hamburg, Germany) and sequence analysis of the *ompA* gene [50, 51] and the 17 kDa protein antigen gene [50] using the AccuPower® ProFi Taq PCR Premix (Bioneer, Daejeon, Korea). The 20 μl reaction mix was composed of 1 μl (10 pmol) of each primer (Table 2), 13 μl of ddH_2_O, and 5 μl of total nucleic acid. The PCR conditions are shown in Table 2. No DNA template was used in negative control.

The sensitivity of rickettsial UR-qPCR was examined using the recombinant DNA of *R. japonica*, a DNA fragment 388 bp long corresponding to the amplicon size of primer pair ITS-F/R was chemically synthesized according to the sequence from position 700,066 to 700,453 of *R. japonica* genome (NCBI accession number CP047359). The DNA fragment consisting of 92 bp of 23S ribosomal RNA gene, 253 bp internal transcribed spacer, 43 bp of 5S ribosomal RNA gene was inserted in the pGEM®-T vector system (Promega, Madison, WI, USA) and used as standard DNA for positive control of UR-qPCR detection. Recombinant DNA was serially 10-fold diluted from 2.72 × 10^8^ to 2.72 × 10^0^ copies/μl, and used for UR-qPCR to identify the minimum copy number that could be detected. PCR was performed in triplicate and a linear regression representing the relationship between initial DNA copy number and cycle threshold (Ct) of amplification was established. The specificity of the UR-qPCR system was also evaluated by assessing cross-detection of DNA from five other tick-borne pathogens (*Anaplasma phagocytophilum*, *Ehrlichia chaffeensis*, *E. canis*, *Toxoplasma gondii*, *Coxiella burnetii*, and *Borrelia burgdorferi*) under the same PCR conditions. Specific amplification was determined by comparing the peak of melting curves using sample DNA with that using Rickettsia recombinant DNA. The UR-qPCR assay was also performed using DNA template of five *Rickettsia* species including *R. japonica, R. roultii,* “*Candidatus* R. longicornii”, *R. monacensis,* and *R. tamurae* to verify the possibility of various *Rickettsia* species detection.

### Detection and phylogenetic analysis of *Rickettsia* from ticks

To screen ticks for *Rickettsia* spp., 10 μl total nucleic acid from each of the five tick pools of adults, nymphs, or larvae of the same species collected from the same site was taken and combined to have 50 μl solution mix; then, 3 μl was used for UR-qPCR. The combined nucleic acid with positive UR-qPCR results was identified and each pool tested individually to identify the exact pool carrying the pathogen; conventional nested PCRs targeting *ompA* and 17 kDa protein antigen genes were used for the detection and sequencing analysis.

After confirming the expected bands of ITS DNA (388 bp) and nested PCR products of *ompA* (532 bp, Table 2) and 17 kDa protein antigen genes (450 bp, Table 2) the PCR products were purified using the QIAquick PCR Purification Kit (QIAGEN, Hilden, Germany) prior to being shipped for sequencing by Macrogen Inc. (Seoul, Korea). The generated sequences were deposited on NCBI with accession number MW916824 (17 kDa protein antigen gene), MW916823 (*ompA* gene), and MW929192 (ITS DNA). The gene sequences were aligned using the Clustal X2 program [52], the overhanging ends were trimmed using BioEdit 7.2 software [53], and phylogenetic tree was constructed using the neighbour-joining method and bootstrapped 1000 times using the MEGA7 software [54].

### Statistical analysis

The tick samples were collected and arranged in pools according to living stages of each species collected from the same site for detection of *Rickettsia* spp. Analysis of the prevalence of *Rickettsia* spp. in the tick pools was done using the minimum infection rate (MIR) that based on the assumption that every positive pool contains only one infected tick. The MIR was calculated using the formula: MIR = [(number of positive pools)/(total number of tested ticks)] × 1000 [55, 56].

## Data Availability

All data generated or analysed during this study are included in this published article. All the nucleotide sequences generated from this study have been deposited and are available in the GenBank database (NCBI accession No.: MW916823 and MW916824).

## Abbreviations

UR-qPCR: Ultra-rapid real-time PCR
ITS: Internal transcribed spacer
MIR: Minimum infection rate
